# Ultraviolet Radiation Influences Perch Selection by a Neotropical Poison-Dart Frog

**DOI:** 10.1371/journal.pone.0051364

**Published:** 2012-12-12

**Authors:** Lee B. Kats, Gary M. Bucciarelli, David E. Schlais, Andrew R. Blaustein, Barbara A. Han

**Affiliations:** 1 Natural Science Division, Pepperdine University, Malibu, California, United States of America; 2 Department of Ecology and Evolutionary Biology, University of California Los Angeles, Los Angeles, California, United States of America; 3 Department of Zoology, Oregon State University, Corvallis, Oregon, United States of America; 4 Odum School of Ecology, University of Georgia, Athens, Georgia, United States of America; University of Sao Paulo, Brazil

## Abstract

Ambient ultraviolet-B radiation can harm amphibian eggs, larvae and adults. However, some amphibians avoid UV-B radiation when given the opportunity. The strawberry poison dart frog, *Oophaga pumilio*, is diurnal and males vocalize throughout the day in light gaps under forest canopies that expose them to solar radiation. Previous studies have demonstrated that males calling from high perches are more successful at mating than those at lower perches. We investigated whether frogs at higher perches receive more ultraviolet-B than those calling from lower perches. We also investigated whether frogs on perches receiving relatively low ultraviolet-B levels maintained their positions for longer compared to individuals calling from perches receiving higher levels of ultraviolet-B. Finally, since it has been hypothesized that some animals utilize levels of UV-A as a visual cue to avoid UV-B damage, we artificially elevated ultraviolet-A levels to examine whether males exposed to artificially elevated ultraviolet-A abandoned their perches sooner compared to males exposed to visible light. We found that frogs called from perches receiving low ultraviolet-B regardless of perch height, and that frogs maintain their positions longer on perches receiving low ultraviolet-B compared to perches receiving even slightly higher ultraviolet-B levels. Exposing the frogs to artificially elevated levels of ultraviolet-A radiation caused males to move off of their perches faster than when they were exposed to a control light source. These experiments suggest that ultraviolet radiation plays an important role in frog behavior related to perch selection, even in rainforests where much of the solar radiation is shielded by the forest canopy.

## Introduction

With growing emphasis on amphibian population declines and extinctions [1] scientists are increasingly focusing on environmental factors that may influence amphibian behavior, ecology and reproduction. It appears that climate change, disease, habitat destruction, the spread of invasive species, and interactions among these factors all contribute to amphibian population declines [2]. Studies using satellite data have indicated that levels of one environmental factor, ultraviolet radiation, have increased in regions of Central America [3]. Given that numerous studies have indicated that ultraviolet-B radiation can be harmful to amphibians (e.g., causing mortality, deformities, and genetic defects that carry over to offspring; reviewed in [4], [5]) the role that UV-B might play in the behavior and ecology of Central American amphibians is of great interest.

While most adult frogs are nocturnal [6], some frogs that are common in the neotropics are diurnal (*e.g.*, poison dart frogs, Dendrobatidae) and likely reflect a long evolutionary history of exposure to ultraviolet radiation influencing ecologically important behavior [7]. For example, a recent study found that two species of neotropical poison-dart frogs (*Oophaga pumilio* and *Dendrobates auratus*) are behaviorally sensitive to UV-B, and avoided ambient levels of UV-B both in the field and in an experimental setting [7]. However, male poison-dart frogs can also be territorial, seeking light gaps and other openings in the forest understory from which they select perches to vocalize for females [8]. Given that male *O. pumilio* (the strawberry poison-dart frog) seem highly sensitive to low levels of UV-B [7], we wanted to further investigate how UV-B might interact with perch selection. Not only do males of this species seek open areas from which to vocalize, but males from higher perches were more successful at mating than those calling from lower perches [9]. It is widely known that sunlight levels have a vertical gradient in tropical rainforests with low levels of sunlight reaching the forest floor and higher levels at mid and upper canopy strata [10]. If UV levels rise with height from the ground (less vegetative ground cover), male frogs might be experiencing a trade-off between increased perch height and elevated UV-B levels. We also investigated whether UV-B levels influenced the length of time a frog maintained position vocalizing on a perch. We hypothesized that if UV-B levels were an important determinant of perch quality, vocalizing male poison-dart frogs would spend longer time on a low UV-B exposed perch than on a perch with higher levels of UV-B exposure. Finally, we investigated how vocalizing male frogs would respond when exposed to increased levels of UV-A radiation (∼315–425 nm). UV-A radiation comprises over 90% of the solar radiation reaching the earth's surface, and visual receptors in animals that can detect UV radiation are most receptive to wavelengths in the UV-A spectrum [11]. Perhaps not surprisingly, amphibians are known to see UV-A [12], [13]. It has been hypothesized that amphibians may use UV-A as a visual signal to avoid UV-B damage [14]. Siddiqi et al [15] noted that *D. pumilio* did not have a visual cone class specialized for UV vision; however, absorption data indicate that *D. pumilio* cones are absorbing in the UV-A range. If UV-A serves as a visual cue for UV-B we hypothesized that calling frogs would leave perches when exposed to artificially elevated levels of UV-A.

## Methods

### Ethics Statement

Animals were observed and experiments were conducted under government permit #19163 from the Ministerio del Ambiente y Energia de Costa Rica. All procedures were approved by the Institutional Animal Care and Use Committee at Pepperdine University under permit #12-0823.

### UV-B and Perch Height

Surveys were conducted in lowland tropical rain forest at La Selva Biological Research Station in Costa Rica in May 2007. Data were collected between 1000–1400 h when ambient UV-B levels in the open were between 9 and 13 µW/cm^2^. Data were obtained for 69 adult *O. pumilio* over a four day period at different locations along eight trails using 500 m to 1000 m of each trail in order to prevent sampling the same individual multiple times. Ambient UV-B levels were measured with a PMA2102 Outdoor UV-B meter (Solar Light Co., Philadelphia, PA, USA; sensor diameter: 24 mm; detectable range of wavelengths: 280–370 nm, with 100-fold higher sensitivity in the UV-B portion of this range). We sampled along trails through secondary growth forest where *O. pumilio* were most abundant.

Adult males were located by walking sections of trails and listening for male frog calls. Only perch locations where frogs were visually confirmed to be calling were measured. When possible, frogs were also captured to obtain snout-vent length measurements and subsequently released. The perch height and the air temperature at each frog's exact location were immediately measured. Perch height is defined as the distance from the ground to the frog perch site.

In addition to measuring UV-B levels at perch sites of vocalizing frogs, we also measured visible light (solar irradiance, 400–1100 nm) for a random subset of frogs using a handheld photometer (LI-COR185B quantum/radiometer/photometer with a pyranometer sensor). We compared levels of UV-B and visible light between calling frogs and randomly determined locations 0.5 m away from each perch site. The direction of the new location was chosen from a list of randomly generated degrees ranging from 0°–360° using a handheld compass with 0° centered on the perch site. At each of these random locations, we measured UV-B and visible light at the same height as the perch site, as well as at 0.25 m intervals between the ground (0 m) and 2.5 m high. Vegetation positioning was left as unaltered as possible when collecting UV-B and visible light measurements, even if vegetation completely obscured the meter at certain locations.

### UV-B and Perch Time

To determine the amount of time males spent vocalizing at perch sites, we walked trails in secondary rainforest searching for vocalizing male *O. pumilio* (2008 and 2009). We observed 65 frogs in 2008, and 29 frogs in 2009. There were no differences between years in time spent on perches (W = 992, p = 0.69), height of perches (W = 963, p = 0.87), or UV-B reaching the frogs (W = 1013, p = 0.56) using Mann-Whitney U tests. Thus, we combined frogs from both years for a total sample size of 94. It is unlikely that the same frog was observed twice since we moved to a different location along the trails, and vocalizing male *O. pumilio* are highly territorial. When a male was located we immediately began timing the length of the calling bout, maintaining enough distance as to not disturb the animal. When the frog moved more than a body length from its initial position we ended the timing of the calling bout. One body length is about the diameter of the UV-B sensor (24 mm). Therefore, we assumed that once a frog moved more than one body length it would be moving into a new UV microhabitat, at least as would be measured by the UV-B sensor. This time does not represent the entirety of each calling bout given we did not capture animals at the start of the bout; however, these data should still indicate long calling periods on preferred perches and shorter times on less preferred perches. In preliminary observations, frogs called for up to 900 s without moving, but we noted interactions between frogs as early as 600 s. For animals that did not stop calling, we therefore set a maximum time on a perch at 600 s, after which we ceased timing. We also measured UV-B at these perch sites as well as at random locations 20 cm away from each perch at a 90° rotation (selected randomly) in the same horizontal plane. This nearby random location allowed us to compare UV levels at the perch to a nearby site. Given previous results showing that male frogs tended to call from perches with low UV-B exposure (0.12±0.02 µW/cm2; [7]), we also compared the time spent calling from perches with UV-B levels above 0.12 µW/cm^2^ to the time spent calling from perches with UV-B levels equal to or below 0.12 µW/cm^2^. If low UV-B exposure is important to perch selection we predicted that frogs would spend more time on perches with low UV compared to those with higher UV. Another previous study showed that the median perch height of calling *O. pumilio* was 26 cm [9]. Thus, we compared the time spent calling from perches above and below 26 cm. If perch height is a key factor in perch selection we predicted that frogs on perches above 26 cm would spend more time on those perches than frogs on perches below 26 cm.

We also conducted a second experiment examining UV and time spent on perch. Experiments were conducted between 1000 h and 1400 h. In this experiment we used the same mylar (transmits about 3% UV-B) and acetate (transmits about 82% UV-B) filters that were used in Han *et al.* [7]. These filters did not differ in transmitted solar irradiance (400–1100 nm) [7]. Filters were mounted on 25 cm×20 cm frames and then attached to a telescoping pole that extended to 2 m. When calling frogs were located either the mylar filter or the acetate filter was slowly extended to cover the frog. Filters were 5–10 cm above the frog. We then timed how long the frog stayed on its perch. Time was stopped when the frog moved at least one body length. Also, at the conclusion of each trial, ambient UV-B was recorded at the perch location. If perch behavior is influenced by UV-B we predicted that frogs covered with mylar filters would stay on perches filter longer than frogs covered with acetate.

### Artificial Elevation of UV-A

To investigate how vocalizing male frogs would respond to elevated UV-A we used a portable, hand-held light (Streamlight Twin-Task 3C-UV) that emitted either UV light (in the UV-A range: 365&390 nm) or visible light (range: 400–780 nm). The UV beam emitted 0.30 µW/cm^2^ at a distance of 15 cm. The visible light emitted a beam that contained wavelengths that crossed into the spectrum read by the handheld photometer (0.05 µW/cm^2^ at a distance of 15 cm). A team of two researchers walked through the rainforest in search of vocalizing dart frogs between 1000 h and 1400 h. When a frog was located one observer held the light approximately 1.5 m from the frog at a randomly chosen condition (light off, visible light on, UV light on). The observer holding the light would then slowly move the light in a direct path toward the calling frog. At the point where the frog moved from its perch, the approaching light was stopped and the second observer used a retractable measuring tape to measure the distance between the frog perch location and the handheld light. If the light beam was located within 1 cm of the frog before the frog moved, 1 cm was recorded.

## Results

### UV-B and Perch Height

**Figure 1:**
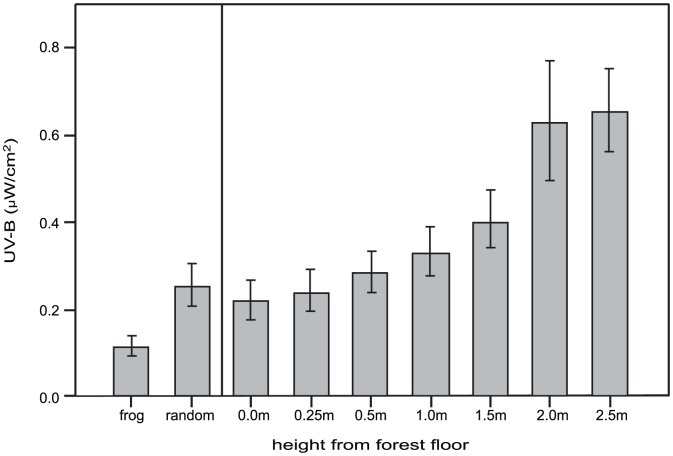
UV-B levels at frog perches and nearby random locations. Comparison of mean UV-B levels (with standard error bars) at the frog perches (frog); at a random location 20 cm from the frog perch at the same height (random); at ground level (0 m), and at six distances above the ground (0.25 m–2.5 m).

**Figure 2:**
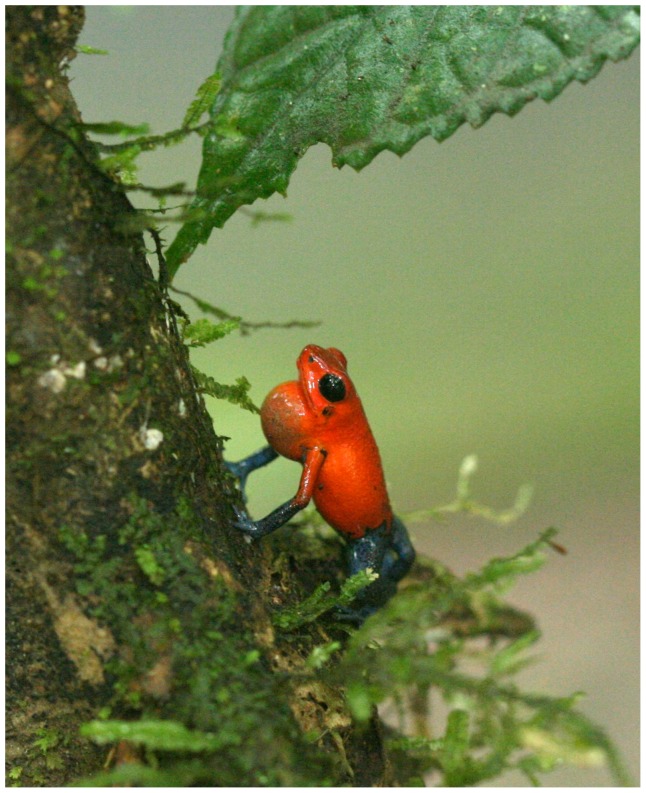
An example of a typical frog perch. Frogs were often seen calling from perches that offered cover from UV-B almost immediately above their location. (Photograph by Silas Dudley).

We recorded the perch height and UV-B from 69 vocalizing male frogs (SVL: 19–24×mm; air temperature: 26.0–31.0°C). The mean perch height was 24.1 cm±2.9 SE. The mean UV-B level at perch sites was 0.12 µW/cm^2^±0.02 SE and the mean UV-B at the nearby random sites at the same height was twice as high, 0.25 µW/cm^2^±0.05 SE. This difference was significant (paired t-test, *t* = 2.89, *P*<0.01, df = 68; Fig. 1). Mean UV-B levels increased with height measured up to 250 cm, with mean UV-B levels ranging from 0.22 µW/cm^2^ (±0.05 SE) on the ground to 0.66 µW/cm^2^ (±0.07 SE) at 250 cm above the ground (Fig. 1). We found that frogs perched above 26 cm were exposed to 0.10 µW/cm^2^±0.03 SE (N = 46) compared to frogs perched at or below 26 cm (0.13 µW/cm^2^±0.03 SE, N = 23). This difference was not significant (*t* = 0.6, *P* = 0.54, df = 67). Mean visible light levels did not differ between perch sites above and below median height (*t* = 0.2, *P* = 0.83; above: 160.4 lumens±18.7 SE, N = 10; below: 154.5 lumens±16.7 SE, N = 22). Frequently frogs were seen on perches that included cover just above their head (Fig. 2).

### UV-B and Perch Time

We recorded the perch times for 94 vocalizing frogs. Frogs maintained position on their perches for 266.8 s (±21.5 SE). Frogs on perches that were receiving 0.12 µw/cm^2^ or less of UV-B radiation maintained their position for 322.6 s (±27.4 SE, N = 53) while those on perches with measured UV-B above 0.12 µw/cm^2^ maintained their position for 194.6 s (±31.3 SE, N = 41). This difference was significant (t-test, *t* = 3.1, *P*<0.01, df = 92). The mean perch height for frogs found in UV-B at or below 0.12 µw/cm^2^ was 35.0 cm (±2.9 SE), while those observed above 0.12 µw/cm^2^ had a mean perch height of 34.1 cm (±3.3 SE). This difference was not significant (*t* = 0.2, *P* = 0.83, df = 92). Frogs calling at a perch height of 26 cm or below stayed on their perches for 254.7 s (±35.8 S.E., N = 40) while those on perches above 26 cm stayed on their perches for 275.7 s (±26.7 S.E., N = 54). This difference was not significant (*t* = 0.56, *P* = 0.63, df = 92).

In the second experiment, vocalizing frogs shielded with mylar (N = 18) vocalized longer (297.2 s±23.7 SE) than frogs shielded with acetate (N = 16; 218.8 s±36.7 SE; t = 1.96, *P* = 0.03, one-tailed, df = 32). Ambient UV-B was not significantly different between the two groups (t = 0.61, *P* = 0.54).

### Artificial Elevation of UV

Frogs responded differently to the approaching light depending on whether the light was emitting UV-A, visible light or was turned off. When the approaching light was emitting UV-A, frogs abandoned their perches when the light was 26.8 cm (±4.9 SE, N = 27) away. When the light was either turned off or emitting visible light, the frogs did not jump until the light was significantly closer (off: 16.7 cm±3.1 SE, N = 25; visible light: 8.7 cm±2.1 SE, N = 23; ANOVA, *F* = 6.0, *P*<0.01, df = 2, 72; p-values for Fisher's PLSD post-hoc comparisons: UV vs. visible light, p<0.01; UV vs. off, p = 0.05; visible vs. off, p = 0.14).

## Discussion

These field observations and experiments support the hypothesis that UV levels influence perch selection by adult male strawberry poison dart frogs. Previously, Han *et al.* [7] demonstrated that adult poison dart frogs avoided high levels of UV-B independently of visible light levels, and were found at levels in the rainforest that were approximately six times lower than random locations in the forest. Our more recent observations on frogs found that they occupied perches in the rainforest at mean UV-B levels identical to those reported by Han *et al.* [7] (0.12 µW/cm^2^). Despite our observations that UV-B and visible light levels climb with height above the forest floor, the frogs consistently occupied locations with much lower levels of UV-B with no difference in visible light. At random locations near frog perches, UV-B levels were double those recorded at the frogs. UV-B levels at 25 cm above the forest floor were also double those at the frog perches. Our data suggest that in random locations throughout the rainforest UV-B levels increase with height. However, vocalizing dart frogs select perch locations at all heights that receive significantly lower levels of UV-B radiation.

Poison dart frogs have long been known to be territorial [8], [16], [17] and return to their original locations when experimentally translocated [18]. Some have suggested that males defend areas that contain resources important for females (*e.g.*, tadpole-rearing sites; [16]), or areas that contain high densities of females [8]. Male frogs may also defend areas to minimize interference from rival males [19], and may compete for areas with favorable sound transmission features [8], [20]. Our study suggests that one feature previously overlooked about poison dart frog territories and perch locations is the level of UV-B radiation. Frogs in our study spent significantly longer time on perches that were exposed to low levels of UV-B than they did on perches receiving higher levels of UV-B. The tendency for a frog to maintain its position on a perch appeared unrelated to the height of the perch since frogs at low UV levels and higher UV levels called from perches with similar mean heights.

If frogs are using UV-A levels as a visual indicator of UV-B or directly sensitive to UV-A, we predicted that artificially elevating UV-A on a calling frog would cause it to abandon its perch. We tested this by exposing a frog to an approaching light that was either off, emitting visible light or emitting UV-A light, and found that frogs abandoned their perches more frequently and at greater distances away from an approaching light (three times greater) when exposed to UV-A light compared to visible light. In contrast, frogs exposed to visible light actually stayed on their perches the longest. It is not clear why frogs in elevated visible light would remain on their perches longer than frogs exposed to an approaching light emitting no beam. Frogs may have been disoriented by the approaching visible light beam or this elevation in visible light may have provided conditions more favorable to the frog. For example, Summers et al. [21] found that female dart frogs use visual cues to select mates and mate choice is apparently based on color perception. Thus, one possibility is that in the visible light treatment of our experiment, frogs may have remained on their perches longer because the additional white light enhanced female perception of their color.

All three of our experiments suggest that poison dart frog perch selection is influenced by levels of UV radiation. It could be that microsites with high UV levels are suboptimal for other reasons that were not considered in our study (e.g., sites were more exposed, or exhibited additional characteristics that co-vary closely with ambient UV levels). However, our observations suggest that frogs do not experience a trade-off with higher UV-B when selecting higher perches. Somehow, frogs find perches with low levels of UV-B regardless of perch height, despite our data on random rainforest locations indicating that UV-B levels rise with increased height from the forest floor. Field data and observations on frog behavior and UV-B may also be correlated with other environmental factors that correspond with UV-B levels. However, the behavioral patterns related to UV levels are further supported in field manipulations where UV is directly manipulated either by filters or by artificial light. Frogs prefer to maintain their position for longer periods of time on perches with low UV-B levels versus perches with high UV-B levels. This is further supported by the tendency of frogs to leave perches when UV-A is experimentally elevated. Unlike most tropical frogs, many poison dart frogs, including *O. pumilio*, are diurnal. Given that amphibians can be susceptible to sublethal effects from UV-B [4], [22], some species protect themselves by increasing melanin [23]. To our knowledge, there is no evidence that *O. pumilio* change melanin levels in response to UV. It is possible that *O. pumilio* may have evolved both physiological and behavioral mechanisms to detect and avoid even low levels of UV-B. While the exact mechanisms used by *O. pumilio* to detect UV-B are not known with certainty, it is known that many vertebrates including some amphibians are able to see in the UV spectrum and our experiment suggests frogs may visually cue in on UV-A. Siddiqi *et al.* [15] concluded that *O. pumilio* did not have cones specialized for UV vision, however, their microspectrophotometric analyses of *O. pumilio* photoreceptors indicate that there is absorption into UV-A wavelengths. Further, they confirm that *O. pumilio* can see color and likely choose mates and interact with conspecifics based on color. We also know that some amphibians use UV-based communication to select mates [24], [25]. We speculate then, that in addition to using visual cues to identify mates, UV vision might be beneficial for mate selection and by enabling frogs to select microsites sheltered from potentially damaging exposure to UV. Furthermore, selection that favors choosing good perches (e.g., those maximizing male vocalizations; [20]) or offering access to more resources [16] may also have favored microsites that exposed calling frogs to minimal UV-B exposure.
